# Integrating Metabolomics and Transcriptomics Reveals the Multi-Target Mechanisms of Fermented *Portulaca oleracea* L. (Purslane) in Attenuating Acute Diarrhea

**DOI:** 10.3390/nu18162595

**Published:** 2026-08-07

**Authors:** Rui Chen, Chunnan Yan, Xiyu Li, Xiaona Pang, Junhua Jin, Hongxing Zhang, Yuanhong Xie

**Affiliations:** 1 Beijing Laboratory of Food Quality and Safety, College of Food Science and Engineering, Beijing University of Agriculture, Beijing 102206, China; 18311423961@163.com (R.C.); yanchunnan777@163.com (C.Y.); xiyuli1995@sina.com (X.L.); pang_xiaona@bua.edu.cn (X.P.); jinjunhua002008@163.com (J.J.); hxzhang51@163.com (H.Z.); 2 National Center of Technology Innovation for Dairy, Hohhot 100118, China

**Keywords:** purslane, probiotic co-fermentation, metabolomic remodeling, acute diarrhea, transcriptomics

## Abstract

**Background:** Current understanding of the biotransformation of bioactive compounds in *Portulaca oleracea* through probiotic fermentation remains limited, and the anti-diarrheal mechanisms of the resulting fermented products have yet to be fully elucidated. **Methods:** In this exploratory study, we employed untargeted metabolomics together with a senna-induced acute diarrhea mouse model to investigate *P. oleracea* co-fermented with *Lactiplantibacillus plantarum* and *Bacillus subtilis*. **Results:** Metabolomic profiling identified 220 metabolites significantly altered by fermentation, predominantly comprising increased levels of lipids, phenylpropanoids, and polyketides. Compared with the unfermented *P. oleracea* control, fermented *P. oleracea* (FP) effectively reduced the diarrhea index, lowered serum levels of inflammatory cytokines (IL-1β, IL-6, TNF-α) and neurotransmitters (5-HT, substance P), and restored Na^+^/K^+^ balance as well as ileal histomorphology. Transcriptomic analysis and RT-qPCR validation further demonstrated that FP modulated the expression of genes involved in focal adhesion, PI3K-Akt, cGMP-PKG, and mineral absorption pathways. **Conclusions:** Fermented *P. oleracea* (FP) alleviates acute diarrhea through multi-target mechanisms. Notably, fermentation endows *P. oleracea* with markedly superior intestinal protective effects relative to the unfermented counterpart. These findings provide preliminary experimental evidence supporting the potential of fermented *P. oleracea* as a candidate for intestinal protective functional food research.

## 1. Introduction

Diarrhea, a prevalent gastrointestinal disorder worldwide, is defined as the passage of three or more loose or watery stools per day. It typically persists for several days and can result in dehydration due to substantial fluid loss. Diarrhea may be infectious in origin, caused by viruses, bacteria, or parasites acquired through contaminated food, water, or contact [1], or non-infectious, linked to factors like lactose intolerance, inflammatory bowel disease, hyperthyroidism, or medication side effects [2]. In addition to adversely affecting patients’ quality of life, diarrhea can swiftly disturb fluid balance and essential electrolytes, including sodium and potassium, which may result in circulatory failure and organ damage [3]. Although there has been a significant decrease in mortality due to the use of oral rehydration salts and zinc supplementation, diarrhea continues to pose a major global public health challenge [4]. Therefore, there is an urgent need for the development of novel safe and edible interventions and the optimization of dietary and functional food strategies for intestinal health management, both of which are of considerable practical importance.

*Portulaca oleracea* L. (purslane) is a widely recognized edible and medicinal homologous plant, consumed globally as a green vegetable and valued for its health-promoting properties. It is a traditional food and medicine homologous plant widely distributed from the eastern Mediterranean to China [5]. According to the Pharmacopoeia of the People’s Republic of China, *P. oleracea* is characterized by a sour taste and cold nature, and used in dietary regimens to support intestinal comfort and relieve digestive discomfort [6]. Modern pharmacological studies have confirmed that *P. oleracea* contains a complex and diverse array of bioactive phytochemicals suitable for functional food development, primarily including flavonoids, alkaloids, terpenoids, fatty acids, vitamins, and minerals [7]. *P. oleracea* flavonoids have been shown to downregulate the phosphorylation level of the PI3K/AKT pathway in vitro, thereby significantly inhibiting the release of nitric oxide and proinflammatory cytokines in RAW264.7 cells [8]. Additionally, four alkaloids isolated from *P. oleracea* were found to suppress the expression of the proinflammatory cytokines IL-1β and TNF-α in LPS-stimulated RAW264.7 cells [9]. In animal models, the aqueous extract of *P. oleracea* alleviated intestinal discomfort and supported gut barrier function in bacterial diarrhea in mice by modulating the gut microbiota, attenuating intestinal inflammatory damage, and restoring intestinal barrier function [10]. Moreover, *P. oleracea* extract exerted anti-inflammatory effects and ameliorated diarrhea with hematochezia by regulating the NF-κB and PPAR-γ pathways and inhibiting cellular apoptosis [11]. Notably, purslane juice exhibited a more pronounced protective effect than the aqueous extract (POE) against dextran sulfate sodium-induced ulcerative colitis, achieved through inhibition of NLRP3 inflammasome-mediated pyroptosis and repair of the intestinal barrier [12]. However, investigations focusing on probiotic-fermented *P. oleracea* remain insufficient and scattered. Available preliminary evidence has demonstrated that fermentation of Portulaca oleracea with lactic acid bacteria markedly elevates the abundance of phenylpropanoids and improves its antioxidant activity [13]. In animal and livestock trials, fermented purslane was found to regulate gut microbiota composition, reduce diarrhea incidence in weaned piglets, and relieve inflammatory lesions by inhibiting NF-κB pathway activation [14,15]. Even so, existing studies merely explored partial in vitro activity or chronic inflammatory models, and few reports have systematically evaluated the therapeutic efficacy of fermented purslane against acute diarrhea. Furthermore, no research has integrated metabolomics and transcriptomics to elaborate its multi-target regulatory mechanisms covering intestinal barrier repair, which creates an urgent research gap to be addressed.

Probiotic fermentation represents a promising green processing technology for enhancing the functionality of edible and medicinal homologous plants, thereby meeting the growing demand for clean-label and high-bioactivity functional foods. This approach employs microbial biotransformation to improve phytochemical availability, sensory properties, and physiological benefits, thus facilitating the industrialization and functional food innovation of traditional edible herbs [16]. Frequently employed fermentative strains include lactic acid bacteria, yeasts, and *Bacillus* spp. In the course of transforming the constituents of Chinese herbal medicines, these strains and their metabolites—such as short-chain fatty acids and bacteriocins—can also modulate the gut microbiota and bolster immunity, thereby achieving a synergistic interaction between the medicinal compounds and the bacteria [17]. For example, the fermentation of red ginseng with *Lactiplantibacillus plantarum* has been shown to increase the concentration of rare ginsenosides, enhance immune function, and alleviate intestinal inflammation. Fermentation mediates deglycosylation of ginsenosides to produce rare ginsenosides, which achieves higher intestinal absorption and improved pharmacokinetic bioavailability [18]. The fermentation of cornelian cherry fruit with *Bifidobacterium* and *Bacillus subtilis* has been shown to significantly increase the gallic acid content, modify the proportion of triterpenoid components, and enhance both antioxidant and immunomodulatory activities [19]. In a similar vein, the fermentation of Danggui Buxue Decoction with *L. plantarum* resulted in increased polysaccharide and flavonoid contents, augmented hypoglycemic activity, and improved insulin resistance [20]. Additionally, the fermentation of rhubarb with *K. marxianus* KM12 facilitated the conversion of conjugated anthraquinones into free anthraquinones, thereby significantly reducing diarrheal toxicity while maintaining anti-inflammatory and laxative properties [21].

The present study aimed to explore the changes in bioactive components and functional properties of *P. oleracea* following probiotic fermentation, as well as its beneficial effects on senna leaf-induced acute intestinal discomfort in mice. Metabolomic analysis was conducted to identify metabolite differences before and after fermentation. A mouse model was subsequently employed to validate the gut-protective and anti-diarrheal functions of fermented *P. oleracea* by assessing intestinal recovery. The underlying mechanisms were further investigated, with a focus on intestinal barrier function, inflammation, ion homeostasis, neurotransmitter levels, and transcriptomic alterations. These findings provide a scientific basis for the development of fermented purslane as a safe, edible functional food ingredient for maintaining intestinal health and alleviating transient diarrhea.

## 2. Materials and Methods

### 2.1. Bacterial Strains and Culture Conditions

*Lactiplantibacillus plantarum* Zhang-LL (CGMCC No. 6939) was isolated from a traditional fermented Chinese meat product, whereas *Bacillus subtilis* SH1 (CGMCC No. 18612) was derived from soybean paste. Both bacterial strains underwent two successive subcultures in MRS and LB liquid media, respectively, and were incubated at 37 °C for 24 h. Following incubation, each strain was centrifuged at 12,000 rpm at 4 °C for 2 min and subsequently washed with an equivalent volume of sterile normal saline, a process repeated three times. The viable cell counts for *L. plantarum* Zhang-LL and *B. subtilis* SH1 were determined using the plate count method. The bacterial suspensions were then adjusted to the desired concentration using sterile normal saline.

### 2.2. Preparation of Fermented Portulaca oleracea L.

One hundred grams of *P. oleracea*, obtained from Yunnan, China, were combined with one liter of ultrapure water and allowed to soak for one hour. Subsequently, the mixture was subjected to boiling for 90 min and then cooled to yield an aqueous decoction of *P. oleracea. L. plantarum* Zhang-LL and *B. subtilis* SH1 were inoculated into this decoction in a 1:1 ratio, with an inoculation concentration of 2.0 × 10^5^ CFU/mL, to establish the fermented *P. oleracea* group (FP). The decoction without inoculation served as the unfermented *P. oleracea* group (UFP). Both groups were incubated at 37 °C for a duration of five days. Triplicate independent fermentation batches were carried out to assess process reproducibility. The final pH of fermented *Portulaca oleracea* was 4.47 ± 0.06 (CV = 1.38%). The viable populations of *L. plantarum* Zhang-LL and *B. subtilis* SH1 were 8.69 ± 0.11 log CFU/mL (CV = 1.27%) and 5.88 ± 0.22 log CFU/mL(CV = 3.69%). The fermentation broth was subsequently centrifuged at 8000 rpm at 4 °C for 10 min, and the supernatant was collected and subjected to freeze-drying. A precise amount of 50 milligrams of the freeze-dried sample was weighed and placed into a 2 mL centrifuge tube, to which a grinding bead and 400 μL of an extraction solvent (methanol-water mixture, 4:1 *v*/*v*) were added. The sample was processed through grinding using a frozen tissue grinder maintained at −10 °C and operating at 50 Hz for a duration of 6 min. This was followed by a low-temperature ultrasonic extraction conducted at 5 °C and 40 kHz for 30 min. Subsequently, the sample was incubated for 30 min at −20 °C and then subjected to centrifugation at 13,000× *g* for 15 min at 4 °C. The supernatant obtained was collected and dried using nitrogen gas. The resulting residue was reconstituted by adding 120 µL of a reconstitution solution, which comprised a 1:1 volume ratio of acetonitrile and water. This mixture was vortexed for 30 s and underwent a second low-temperature ultrasonic extraction at 5 °C and 40 kHz for 5 min. A subsequent centrifugation was performed at 13,000× *g* for 15 min at 4 °C, after which the supernatant was collected for further analysis.

### 2.3. Untargeted Metabolomics Analysis

The LC-MS analysis was performed by Shanghai Majorbio Biomedical Technology Co., Ltd. (Shanghai, China). The ultra-high performance liquid chromatography–Fourier transform mass spectrometry system (UHPLC-QExactive, Thermo Fisher, Wilmington, NC, USA) was utilized for LC-MS analysis. Chromatographic separation was achieved using an ACQUITY UPLC BEH C18 column (100 mm × 2.1 mm, inner diameter 1.7 µm, Waters, Milford, CT, USA) maintained at a temperature of 40 °C. The mobile phase consisted of two components: mobile phase A, which was a 2% acetonitrile aqueous solution with 0.1% formic acid, and mobile phase B, an acetonitrile solution containing 0.1% formic acid. The injection volume was set at 3 µL. Mass spectrometry analysis was conducted using an electrospray ionization (ESI) source, with data acquisition performed in both positive and negative ion scanning modes. Metabolite identification was achieved by matching the accurate precursor ion masses (MS1) and MS/MS fragmentation spectra against the MagiBio in-house plant-specific database (MJDBPM) as well as public databases including HMDB. The identification confidence level was assigned according to the criteria of the Metabolomics Standards Initiative (MSI).

### 2.4. Animal Experiment Design and Drug Administration

Male Kunming mice of specific pathogen-free (SPF) grade, with an average weight of 20 ± 2 g, were acclimatized for a duration of seven days under pathogen-free conditions, with unrestricted access to food and water. Prior to the initiation of the experimental procedures, the mice were subjected to a fasting period of 8 to 10 h. During the experimental phase, all mice, except those in the control group, received an intragastric administration of 0.2 mL of senna leaf concentrate at a concentration of 1 g·mL^−1^. Two hours following the senna leaf gavage, drug administration was initiated as part of the treatment protocol. Based on previous studies in this field, mice were randomly assigned to nine groups using a computer-generated random number table, each consisting of six mice. Group 1 served as the control group, while Group 2 functioned as the model group and received 0.2 mL of normal saline. Group 3 was administered a pretreatment of 0.2 mL of loperamide at a concentration of 0.5 mg·mL^−1^, serving as the positive control. Groups 4, 5, and 6 received 0.2 mL of unfermented *P. oleracea* at low (UFP-L, 0.5 g·mL^−1^), medium (UFP-M, 1 g·mL^−1^), and high (UFP-H, 2 g·mL^−1^) doses, respectively. Similarly, Groups 7, 8, and 9 received 0.2 mL of fermented *P. oleracea* at low (FP-L, 0.5 g·mL^−1^), medium (FP-M, 1 g·mL^−1^), and high (FP-H, 2 g·mL^−1^) doses, respectively. After administering the drug, animal defecation was monitored for four hours using filter paper under each cage to evaluate fecal traits and calculate the diarrhea index. At the end of the experiment, three mice were randomly selected from each group, euthanized, and blood samples were collected. The blood was then centrifuged at 3000 rpm for 15 min at 4 °C to obtain serum for further analysis. Simultaneously, the jejunum, ileum, and colon were quickly removed; one part was frozen at −80 °C, and the other was fixed in 4% paraformaldehyde for histology. All animal experiments in this study were reviewed and approved by the Institutional Review Board of Beijing Guzi Testing Technology Co., Ltd. of Committee (GSCS-2026-001). All animal procedures were performed in strict accordance with the Guide for the Care and Use of Laboratory Animals and the ARRIVE 2.0 guidelines, following the 3R principles (Replacement, Reduction, Refinement) to minimize animal suffering.

### 2.5. Histological Analysis of Intestinal Tissues

The small intestinal tissues were preserved in 4% paraformaldehyde for a minimum duration of 24 h, followed by staining with hematoxylin and eosin (H&E). Histological examination was conducted utilizing a microscope. Quantitative analysis of villus height and crypt depth was achieved by assessing a minimum of 10 randomly selected fields per slide.

### 2.6. Measurement of Ammonia and Skatole in Intestinal Contents

Ammonia concentration was quantified in accordance with the standardized protocol described in GB/T 18204.25-2000 [22]. Briefly, 0.1 g of mouse intestinal content was weighed and transferred into a test tube, followed by the addition of 5 mL of 0.005 mol·L^−1^ sulfuric acid absorption solution. The mixture was diluted to a total volume of 10 mL with deionized water. After thorough vortexing, the sample was centrifuged at 6000 rpm for 10 min at 4 °C. A 1 mL aliquot of the supernatant was then diluted with 9 mL of the absorption solution, after which 0.1 mL of 0.5 g·mL^−1^ potassium sodium tartrate solution and 0.5 mL of Nessler’s reagent (KS396540, Yuanye Bio-Technology Co., Ltd., Shanghai, China) were sequentially added. The mixture was allowed to stand for 10 min at ambient temperature, and the absorbance was measured at 425 nm (OD_425_ nm).

Skatole concentration was determined according to a previously described method [23]. Mouse intestinal contents were thoroughly dissolved in 0.01 mol·L^−1^ phosphate-buffered saline (PBS) at a pH of 7.0, and subsequently subjected to centrifugation at 1000× *g* for 10 min at 4 °C. The resulting supernatant was collected. A 100 μL aliquot of this supernatant was then mixed with 142 μL of a chromogenic reagent, which comprised p-dimethylaminobenzaldehyde, ethanol, and sulfuric acid, in a 96-well plate. The absorbance was immediately measured at a wavelength of 580 nm (OD_580_ nm).

### 2.7. Determination of Inflammatory Factors

Serum concentrations of interleukin-1β (IL-1β, FY2040-A), interleukin-6 (IL-6, FY2163-A), and tumor necrosis factor-alpha (TNF-α, FY2132-A) were measured using ELISA kits provided by Beijing Changhua Zhicheng Technology Co., Ltd., Beijing, China.

### 2.8. Quantification of Neurotransmitter

Serum levels of 5-hydroxytryptamine (5-HT, FY2443-B) and substance P (SP, FY2445-B) were determined using commercial assay kits supplied by Beijing Changhua Zhicheng Technology Co., Ltd., Beijing, China.

### 2.9. Quantification of Serum Electrolytes

Serum concentrations of potassium (K^+^, C001-2-1) and sodium (Na^+^, C002-2-1) ions were measured using commercial assay kits from Nanjing Jiancheng Bioengineering Institute Co., Ltd., Nanjing, China.

### 2.10. Association Analysis of Differential Metabolites with Diarrhea-Related Indicators

Spearman rank correlation analysis was performed to explore the associations between differential metabolites before and after fermentation and serum and diarrhea-related physicochemical indicators. All statistical analyses were conducted on the Majorbio Bio-Cloud Platform (https://cloud.majorbio.com). The psych package in R (version 4.1.0) was used to calculate Spearman correlation coefficients and *p*-values, and the pheatmap package was employed to generate the correlation heatmap. The correlation coefficient threshold was set at |r| > 0.6, with a significance level of *p* < 0.05.

### 2.11. Transcriptome Sequencing and Analysis

Ileal tissue samples from control (C), model (M), and fermented *P. oleracea* (FP) groups of mice underwent transcriptome sequencing by Shanghai Majorbio Biomedical Technology Co., Ltd. Key steps included extracting total RNA using the 2 × CTAB method, assessing RNA concentration and purity with a Nanodrop 2000c (Thermo Fisher Scientific, Waltham, MA, USA), and evaluating RNA integrity with agarose gel electrophoresis and an Agilent 5300 Bioanalyzer (Agilent Technologies, Santa Clara, CA, USA). mRNA was isolated from total RNA using oligo(dT)-coated magnetic beads. The isolated mRNA was fragmented into ~300 base pair pieces in a lysis buffer. Single-stranded cDNA was synthesized using reverse transcriptase and random primers, followed by the creation of double-stranded cDNA. These were then end-repaired, purified, and size-selected. Suitable fragments were amplified via PCR to build a transcriptome sequencing library, which was sequenced on the Illumina NovaSeq X Plus platform (Illumina, San Diego, CA USA). Quality control and data analysis were performed on the Majorbio Cloud Platform (https://cloud.majorbio.com).

### 2.12. Reverse Transcription-Quantitative Real-Time PCR (RT-qPCR)

Total RNA was extracted from small intestinal tissues utilizing the Tissue RNA Kit (DP430, TIANGEN Biotech Co., Ltd., Beijing, China). Reverse transcription and quantitative PCR were conducted using the FastKing One-Step RT-qPCR Kit (FP313-01, TIANGEN Biotech Co., Ltd., Beijing, China). RNA concentration and purity were assessed with a Nanodrop 2000c Spectrophotometer (Thermo Fisher Scientific, Waltham, MA, USA). RT-qPCR analysis was executed on the Applied Biosystems 7500 Fast Real-Time PCR System (Thermo Fisher Scientific, Foster City, CA, USA), following the thermal cycling protocol outlined in a previous study [24]. Relative gene expression levels were quantified using the 2^−ΔΔCT^ method, with GAPDH employed as the internal reference gene. Melting curve analysis confirmed a single product for each primer pair. The primers utilized in this study are detailed in Table 1.

## 3. Results

### 3.1. Fermentation-Induced Metabolic Remodeling in P. oleracea

**Figure 1:**
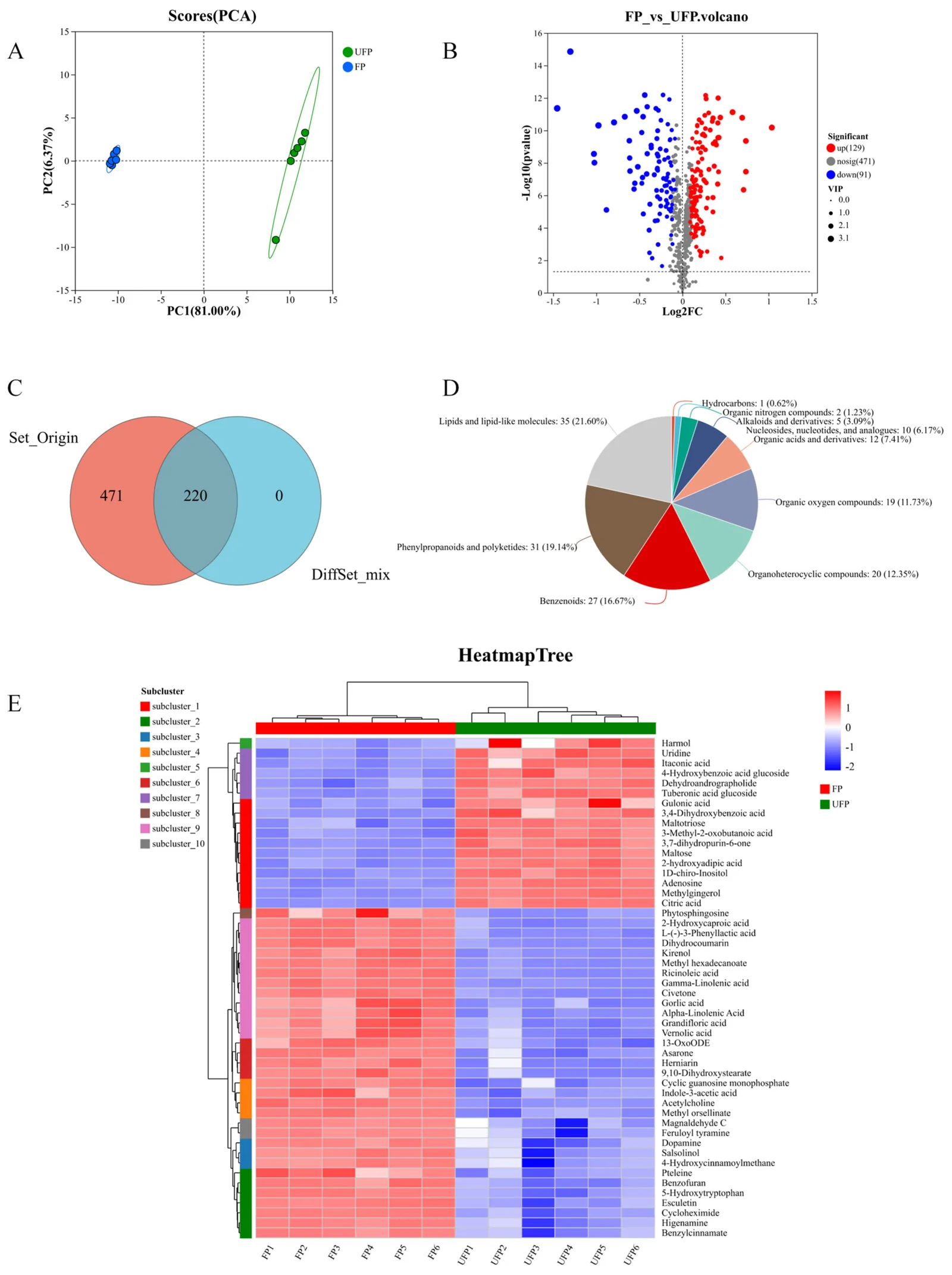
Metabolomic profiling and differential analysis of fermented (FP) and unfermented (UFP) *P. oleracea.* (**A**) Principal component analysis (PCA) score plot depicting the metabolic separation and clustering trends between pre- and post-fermentation samples. (**B**) Volcano plot illustrating significant metabolite alterations in response to fermentation, with red and blue dots indicating up- and down-regulated metabolites, respectively. (**C**) Venn diagram delineating the distribution of shared and unique metabolites between FP and UFP groups. (**D**) Hierarchical clustering heatmap (HMP) depicting the classification and relative abundance of differentially expressed metabolites across 10 chemical categories. (**E**) Heatmap showing expression abundance and hierarchical clustering of differentially accumulated metabolites; color gradient represents z-score-normalized intensity (blue: low abundance; red: high abundance), and dendrograms indicate sample similarity.

A comprehensive metabolomic analysis identified 691 metabolites in both UFP and FP samples. Principal component analysis (PCA) revealed tight clustering within each group, confirming high intra-group reproducibility. Notably, significant separation was observed between the UFP and FP groups. The first principal component (PC1) accounted for 81.00% of the variance, and the second (PC2) for 6.37%, yielding a cumulative contribution of 87.37%, indicating substantial alterations in metabolite composition between the two groups (Figure 1A). Volcano plot analysis identified 220 metabolites with significantly altered abundance between the UFP and FP groups, comprising 129 up-regulated and 91 down-regulated metabolites (*p* < 0.05 and |log_2_FC| > 1, FDR < 0.05). The remaining 471 metabolites showed no significant differences (Figure 1B). The Venn diagram further confirmed these differential patterns, showing 220 altered and 471 unchanged metabolites following fermentation (Figure 1C). High-throughput Differential Metabolomics Analysis (HDME) categorized the 220 differential metabolites into 10 chemical classes (Figure 1D). Lipids and lipid-like molecules were the most abundant (35 species, 21.60%), followed by phenylpropanoids and polyketides (31 species, 19.14%), and benzene ring compounds (27 species, 16.67%). These three categories collectively accounted for over one-third of the total, indicating pronounced activity in related metabolic pathways. Other notable categories included organic oxides (19 species, 11.73%), heterocyclic compounds (20 species, 12.35%), organic acids and derivatives (12 species, 7.41%), and nucleosides and analogs (10 species, 6.17%). Alkaloids and complexes (3.03%), organic nitrogen compounds (1.29%), and hydrocarbons (0.63%) were present in lesser quantities. Cluster heatmap analysis revealed that FP was enriched in lipids, phenylpropanoids, and polyketides. Key lipids included γ-linolenic acid, α-linolenic acid, and phytosphingosine; notable phenylpropanoids included methyl gingerol, esculetin, and feruloyl tyramine; and polyketides such as muscone and methyl orsellinate were also detected (Figure 1E). These results suggest that fermented *P. oleracea* is characterized by enhanced metabolism of unsaturated fatty acids and the synthesis of phenylpropanoid and polyketide metabolites.

### 3.2. Fermented P. oleracea Alleviates Senna-Induced Acute Diarrhea

**Figure 2:**
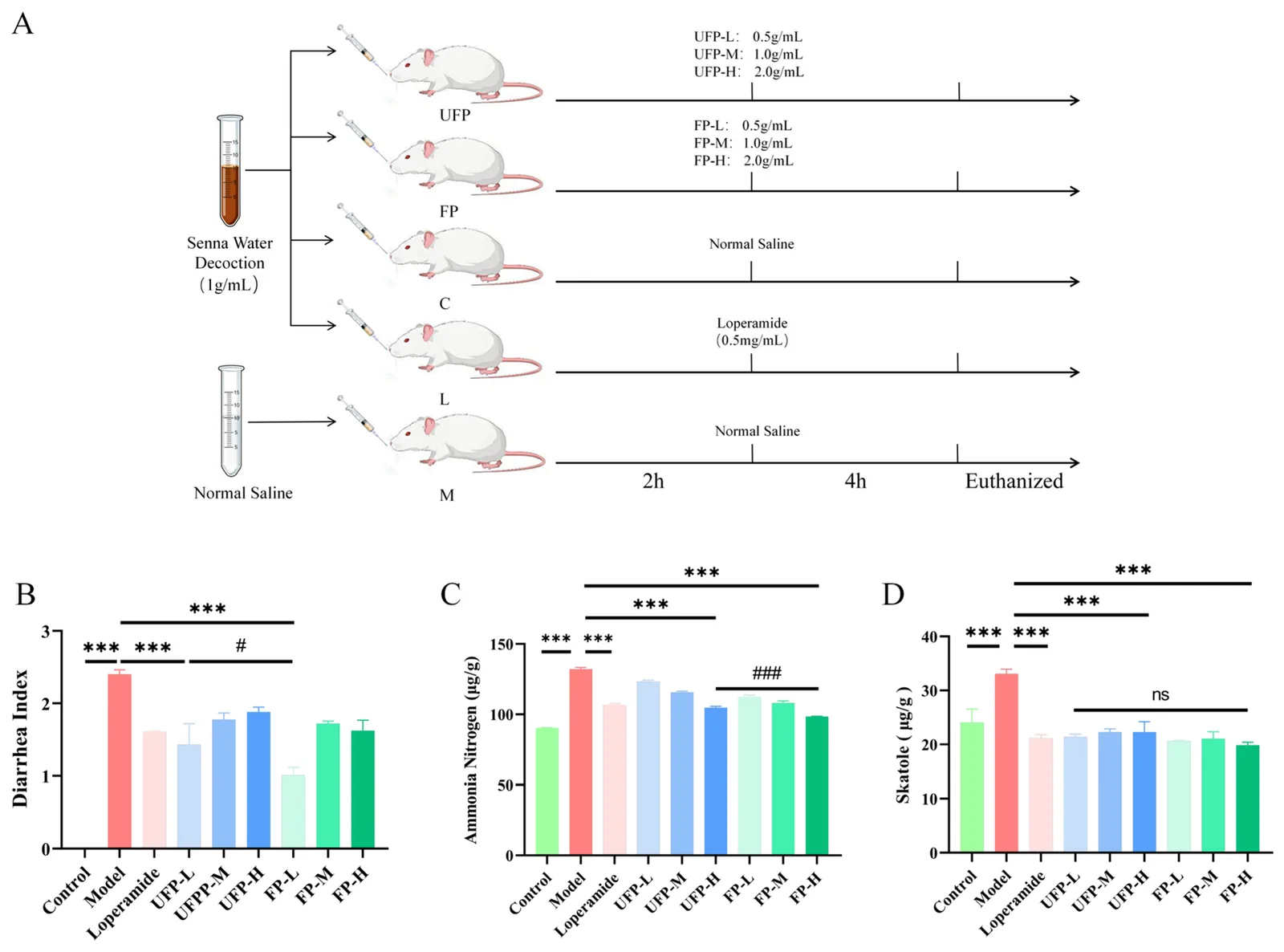
Effects of fermented *P. oleracea* (FP) and unfermented *P. oleracea* (UFP) on diarrhea in mice. (**A**) Schematic illustration of the experimental protocol for acute diarrhea induction and treatment. (**B**) Diarrhea index scores across groups, reflecting anti-diarrheal efficacy. (**C**,**D**) Quantification of intestinal contents showing ammonia nitrogen and skatole levels. Data are expressed as mean ± SD (*n* = 3). *** *p* < 0.001 vs. model group; # *p* < 0.05, ### *p* < 0.001 vs. UFP group. ns, nonsignificant (*p* > 0.05).

This study investigated the in vivo protective effects of UFP and FP on senna-induced acute diarrhea in mice (Figure 2A). The diarrhea index in the model group was significantly elevated (2.40 ± 0.06) compared to the control (*p* < 0.001). Treatment with the positive control drug decreased the diarrhea index to 1.61 ± 0.01, which was significantly lower than that of the model group (*p* < 0.001). Among the various dosage groups of UFP and FP, the low-dose group exhibited the most pronounced therapeutic effect. Specifically, the UFP-L group reduced the diarrhea index to 1.43 ± 0.29, while the FP-L group demonstrated an even more substantial therapeutic effect, further decreasing the diarrhea index to 1.01 ± 0.01 (*p* < 0.001, Figure 2B).

Simultaneously, the ammonia nitrogen concentration in the control group was measured at 90.34 ± 0.22 µg·g^−1^, whereas the model group exhibited a significantly elevated concentration of 132.08 ± 1.24 µg·g^−1^ (*p* < 0.001). Following intervention with the positive drug, the ammonia nitrogen concentration decreased to 106.80 ± 1.02 µg·g^−1^, which exhibited statistically significant differences compared with the model group (*p* < 0.001). Among the various dosage groups of UFP and FP, the high-dose interventions demonstrated the most pronounced effects: the UFP-H group reduced the concentration to 104.71 ± 1.02 µg·g^−1^, and the FP-H group further decreased it to 98.39 ± 0.33 µg·g^−1^. Both reductions were statistically significant compared to the model group (*p* < 0.001), with the FP-H group showing a superior ameliorative effect compared to the UFP-H group (Figure 2C).

In the control group, the skatole content was measured at 24.08 ± 2.47 µg·g^−1^, whereas the model group exhibited a significantly elevated level of 33.08 ± 0.89 µg·g^−1^ (*p* < 0.001). Following intervention with the positive drug, the skatole content was reduced to 21.15 ± 0.71 µg·g^−1^, which exhibited statistically significant differences compared with the model group (*p* < 0.001). Subsequent dose-effect analysis indicated that the low dose of UFP (UFP-L group) was most effective, decreasing the skatole content to 21.43 ± 0.49 µg·g^−1^. Conversely, the high dose of FP (FP-H group) demonstrated superior efficacy, reducing the skatole content to 19.96 ± 0.55 µg·g^−1^, both of which were significantly lower than the levels observed in the model group (*p* < 0.001). Furthermore, the FP-H group showed a superior ameliorative effect compared to the UFP-L group (Figure 2D). These findings confirm that fermented *P. oleracea* effectively ameliorates senna-induced diarrhea symptoms in mice, decreases intestinal ammonia nitrogen and skatole levels, and provides a protective effect on the intestine.

### 3.3. Fermented P. oleracea Restores Intestinal Architecture

**Figure 3:**
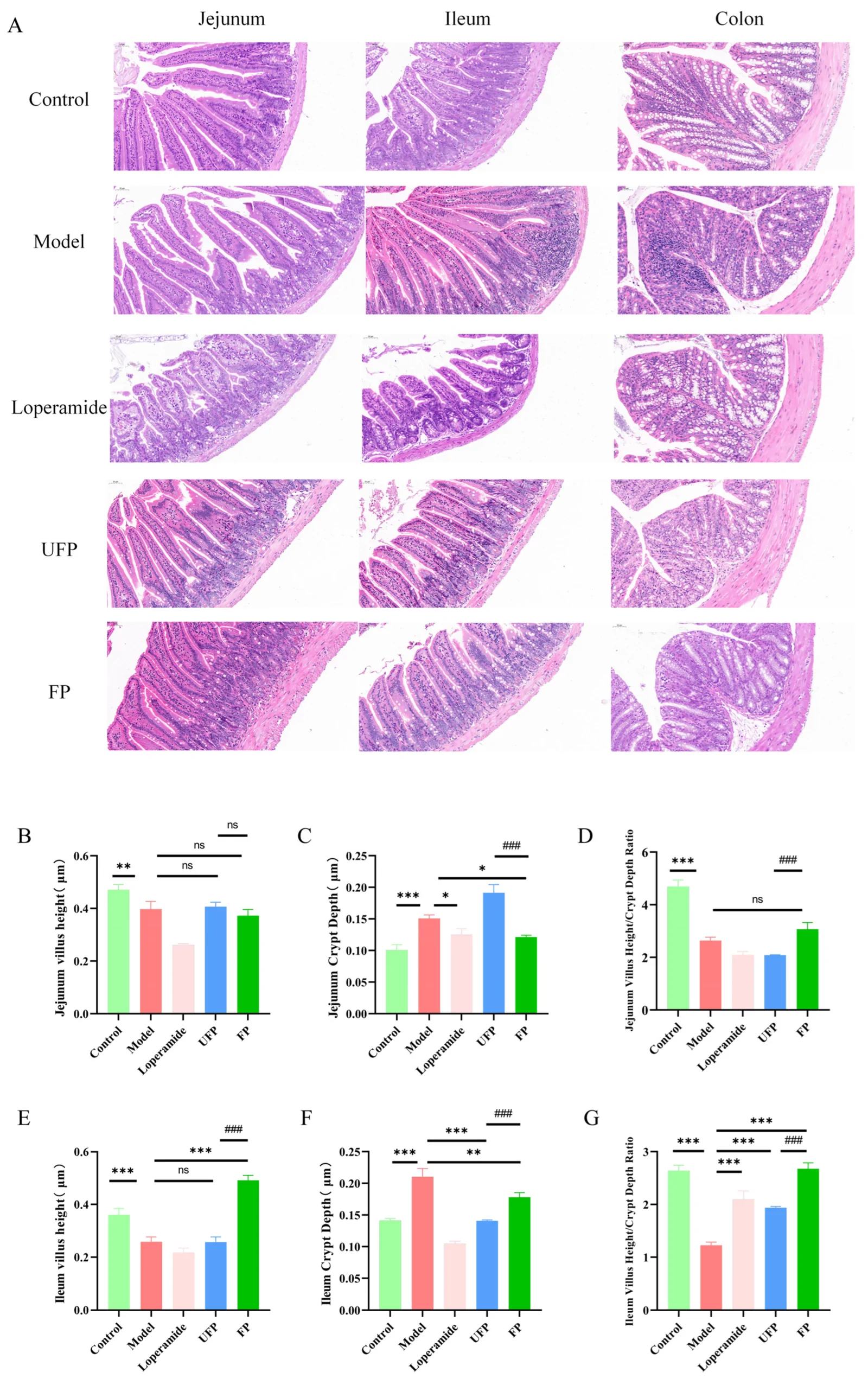
Effects of UFP and FP on intestinal morphology in diarrheal Mice. (**A**) Representative hematoxylin and eosin (H&E) staining images of intestinal tissues (20 × magnification). (**B**,**E**) Quantitative analysis of villus height, (**C**,**F**) crypt depth, (**D**,**G**) the villus height-to-crypt depth ratio (V/C) in the jejunum (**B**–**D**) and ileum (**E**–**G**). At least 10 random fields were measured per section. Data are expressed as mean ± SD (*n* = 3). * *p* < 0.05, ** *p* < 0.01, *** *p* < 0.001 vs. model group; ### *p* < 0.001 vs. UFP group. ns, nonsignificant (*p* > 0.05).

Histological analysis revealed inflammatory damage in the small intestines of model mice, characterized by disordered mucosal structure, atrophic and fused villi, extensive lymphocyte infiltration in the lamina propria, and reduced numbers of goblet cells. Treatment with UFP and FP improved these conditions, restoring villi and reducing lymphocyte infiltration, indicating a protective effect on the small intestine (Figure 3A). To evaluate changes in the small intestinal mucosa, villus height, crypt depth, and the villus-to-crypt (V/C) ratio were measured and analyzed in the jejunum and ileum. Compared to normal tissues, the model group showed reduced villus height, increased crypt depth, and a lower V/C ratio. In the jejunum, villus height in the UFP and FP groups did not differ significantly from the model group (Figure 3B). However, the FP group demonstrated a significant restoration of jejunal crypt depth, whereas the UFP group did not show a significant difference from the model group (Figure 3C). No significant differences in the V/C ratio were observed among the UFP, FP and model groups (Figure 3D). In the ileum, both the UFP and FP groups significantly restored villus height (Figure 3E), reduced crypt depth (Figure 3F), and increased the V/C ratio significantly (*p* < 0.001, Figure 3G). These findings suggested that administration of UFP and FP effectively ameliorated the structural damage to the small intestinal mucosa in diarrheal model mice, particularly in the ileum, thereby enhancing the integrity of the small intestinal mucosal barrier.

### 3.4. Fermented P. oleracea Modulates Pro-Inflammatory Cytokine Release

**Figure 4:**
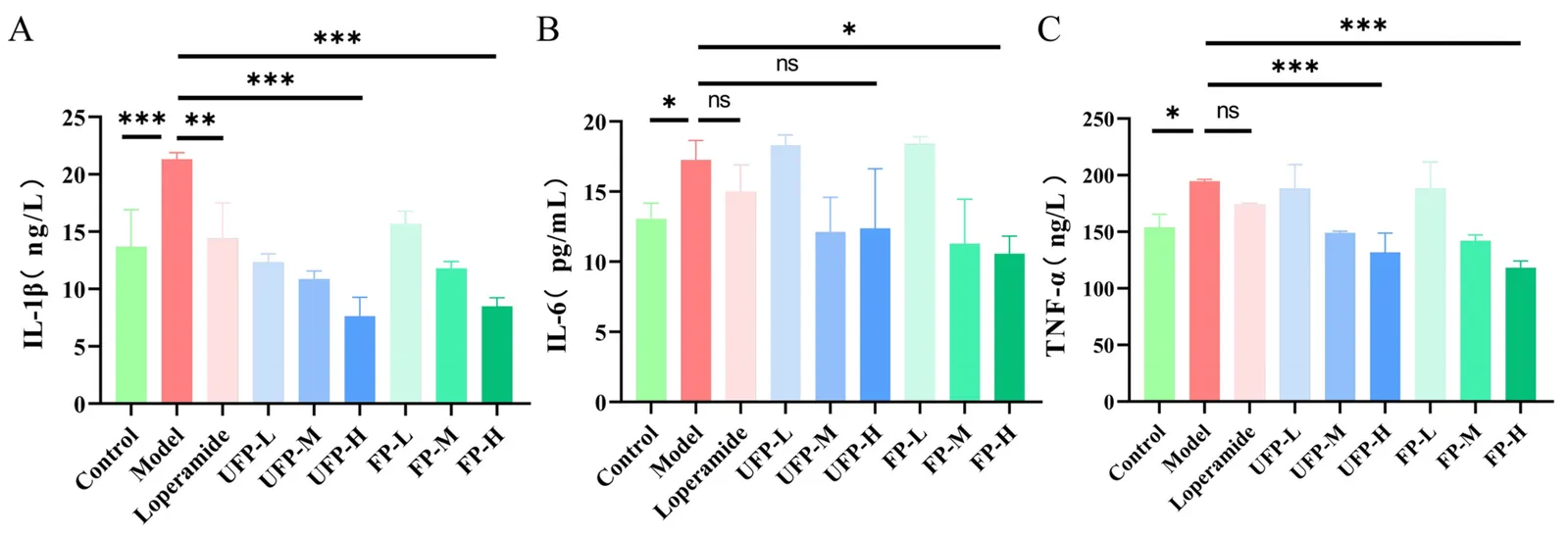
Effects of FP and UFP on serum inflammatory cytokine levels in diarrheal mice. (**A**) IL-1β; (**B**) IL-6; (**C**) TNF-α. Data are expressed as mean ± SD (*n* = 3); * *p* < 0.05, ** *p* < 0.01, *** *p* < 0.001 vs. model group. ns, nonsignificant (*p* > 0.05).

The analysis of serum inflammatory factors in mice revealed that, in comparison to the normal group, the senna-induced diarrhea model group exhibited elevated serum levels of IL-1β, IL-6, and TNF-α, measuring 21.33 ± 0.57 ng·L^−1^ (*p* < 0.001), 17.25 ± 1.38 ng·L^−1^, and 194.62 ± 1.83 ng·L^−1^ (*p* < 0.05), respectively. Following the administration of the positive drug, a significant reduction in serum IL-1β levels was observed, decreasing to 14.41 ± 3.09 ng·L^−1^ (*p* < 0.01) compared to the model group. However, the reductions in serum IL-6 (15.02 ± 1.90 ng·L^−1^) and TNF-α (174.00 ± 1.61 ng·L^−1^) levels were not statistically significant. Among the various dosage intervention groups of UFP and FP, the high-dose group demonstrated the most pronounced effect. Specifically, the UFP-H group reduced the IL-1β level to 7.63 ± 1.65 ng·L^−1^, and the FP-H group reduced it to 8.49 ± 0.75 ng·L^−1^, both of which were significantly decreased compared with the model group (*p* < 0.001, Figure 4A).

Compared to the IL-6 levels observed in the model group, the UFP-H group showed a reduction to 12.37 ± 4.25 ng·L^−1^, which was not statistically significant, while the FP-H group demonstrated a reduction to 10.57 ± 1.26 ng·L^−1^, achieving statistical significance (*p* < 0.05, Figure 4B). In terms of TNF-α levels, the UFP-H group reduced the concentration to 131.78 ± 16.98 ng·L^−1^, and the FP-H group further decreased it to 118.20 ± 6.13 ng·L^−1^, with both reductions being statistically significant (*p* < 0.001, Figure 4C). These results indicate that both UFP and FP effectively reduced serum levels of IL-1β, IL-6, and TNF-α in senna-induced diarrheal mice, with FP demonstrating a more substantial anti-inflammatory effect.

### 3.5. Fermented P. oleracea Reduces Neurotransmitter Levels

**Figure 5:**
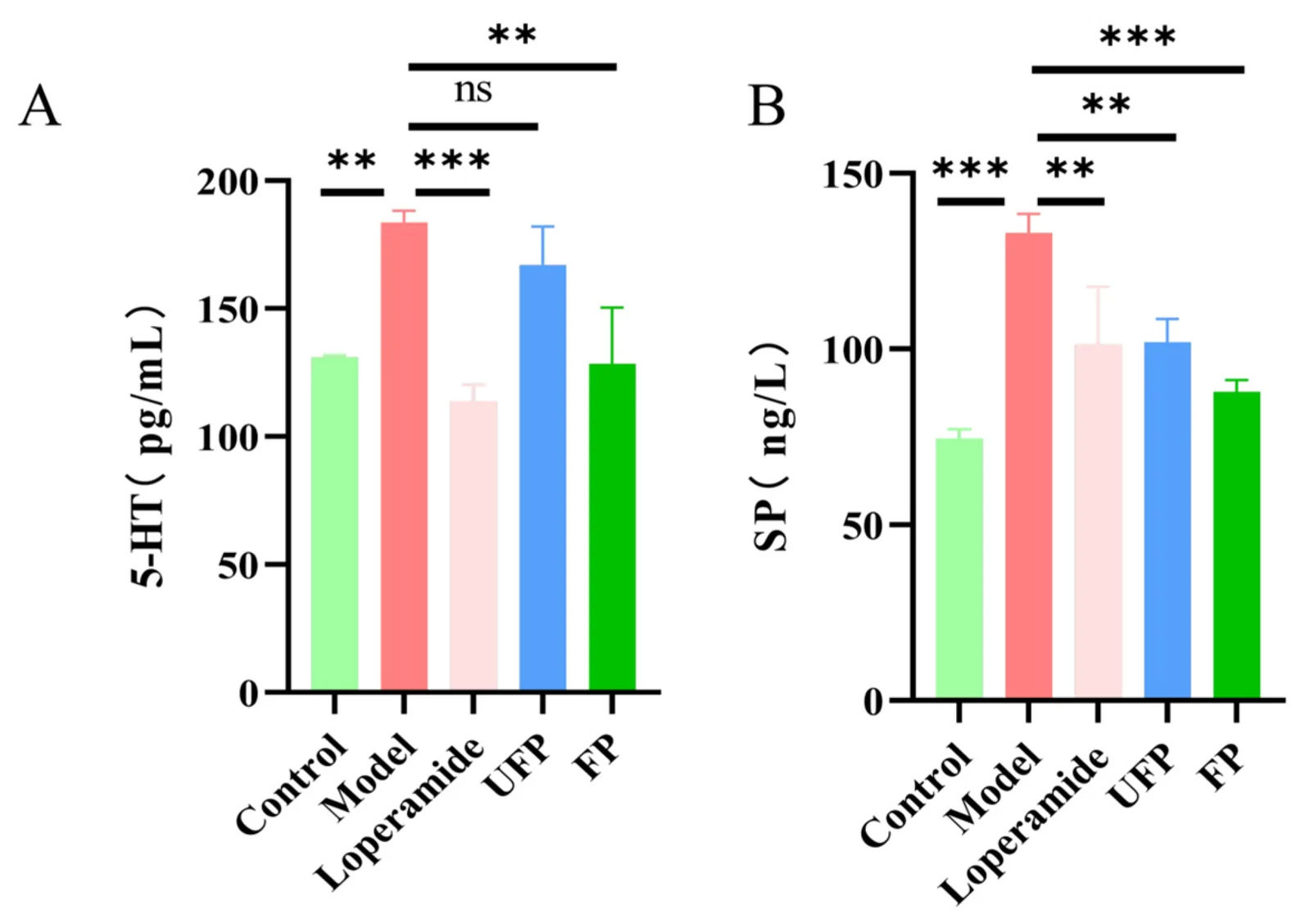
Effects of FP and UFP on serum neurotransmitter levels in diarrheal mice. (**A**) Serum 5-hydroxytryptamine (5-HT) levels. (**B**) Serum substance P (SP) levels. Data are expressed as mean ± SD, *n* = 3; ** *p* < 0.01, *** *p* < 0.001 vs. model group. ns, nonsignificant (*p* > 0.05).

In the model group, the serum 5-HT concentration was significantly increased to 183.82 ± 4.48 ng·mL^−1^ compared with that in the control group (130.87 ± 0.92 ng·mL^−1^) (*p* < 0.01). Compared with the model group, treatment with the positive drug significantly reduced the 5-HT concentration to 113.76 ± 6.27 ng·mL^−1^ (*p* < 0.01), and the FP-treated group also showed a significant decrease to 119.81 ± 17.08 ng·mL^−1^ (*p* < 0.01), whereas the UFP group showed a lower level (167.01 ± 15.03 ng·mL^−1^) that was not statistically significant (Figure 5A). Substance P levels were significantly elevated in the model group (133.11 ± 5.32 pg·mL^−1^) compared to the control group (74.60 ± 2.63 pg·mL^−1^) (*p* < 0.001). Following treatment, all intervention groups exhibited marked reductions: the positive control group decreased to 101.36 ± 16.36 pg·mL^−1^ (*p* < 0.01), the UFP group to 101.97 ± 6.64 pg·mL^−1^ (*p* < 0.01), and the FP group to 87.86 ± 3.31 pg·mL^−1^ (*p* < 0.001, Figure 5B). These findings demonstrate that fermented *P. oleracea* effectively attenuates the elevated serum levels of substance P, restoring them toward control values.

### 3.6. Fermented P. oleracea Corrects Sodium and Potassium Imbalances

**Figure 6:**
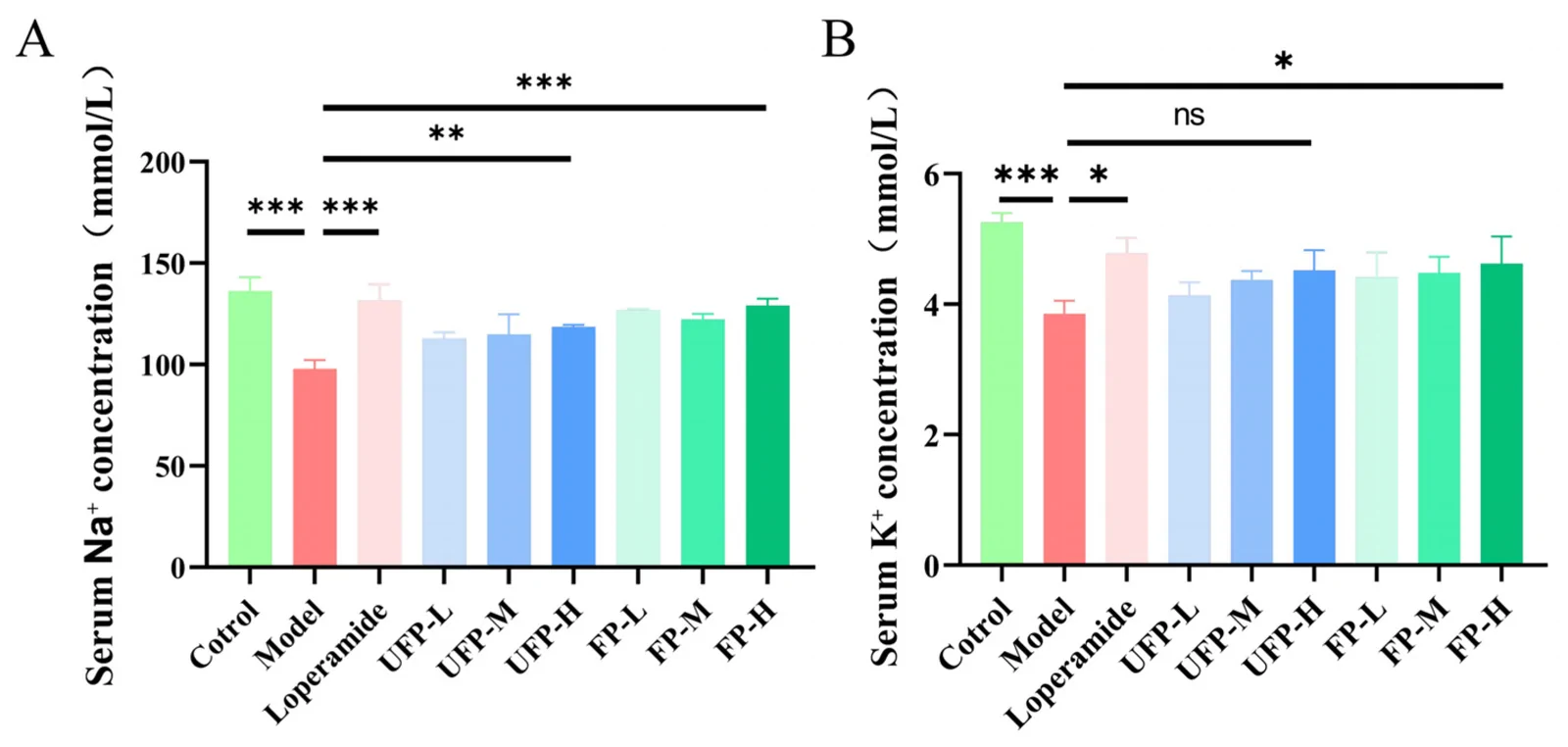
Effects of FP and UFP on serum electrolyte levels in diarrheal mice. (**A**) Serum sodium ion (Na^+^) concentrations. (**B**) Serum potassium ion (K^+^) concentrations. Data are expressed as mean ± SD (*n* = 3); * *p* < 0.05, ** *p* < 0.01, *** *p* < 0.001 vs. model group. ns, nonsignificant (*p* > 0.05).

Compared with the control group, serum sodium ion (Na^+^) concentrations in the model group were significantly decreased (*p* < 0.001). Following administration of the positive drug, the serum Na^+^ concentration increased to 131.65 ± 7.92 mmol·L^−1^, demonstrating significant differences from those observed in the model group (*p* < 0.001). Among the various dosage groups of UFP and FP, the high-dose interventions yielded the most pronounced effects. Specifically, the serum Na+ levels in the UFP-H and FP-H groups increased from 97.83 ± 4.44 mmol·L^−1^ (model group) to 118.59 ± 0.92 mmol·L^−1^ (*p* < 0.01) and 129.14 ± 3.34 mmol·L^−1^ (*p* < 0.001), respectively, both of which were significantly elevated compared to the model group (Figure 6A).

Compared with the control group, the serum concentrations of K^+^ in mice of the model group were significantly decreased (*p* < 0.001). Following administration of the positive drug, the serum K^+^ concentration rose to 4.78 ± 0.23 mmol·L^−1^ (*p* < 0.05). Regarding the regulation of K^+^, the serum K^+^ concentration in the UFP-H group increased from 3.85 ± 0.20 mmol·L^−1^ (model group) to 4.52 ± 0.31 mmol·L^−1^, showing no statistically significant difference compared to the model group. In contrast, the FP-H group exhibited an increase to 4.62 ± 0.41 mmol·L^−1^, which was significantly different from the model group (*p* < 0.05, Figure 6B). These findings suggest that FP was more effective than UFP in restoring ion levels. Both UFP and FP interventions were capable of ameliorating diarrhea-induced electrolyte imbalances to varying extents, with the high dose of FP demonstrating superior efficacy in reestablishing Na^+^ and K^+^ homeostasis.

### 3.7. Association Analysis of Differential Metabolites with Intestinal Function and Phenotypic Indicators

**Figure 7:**
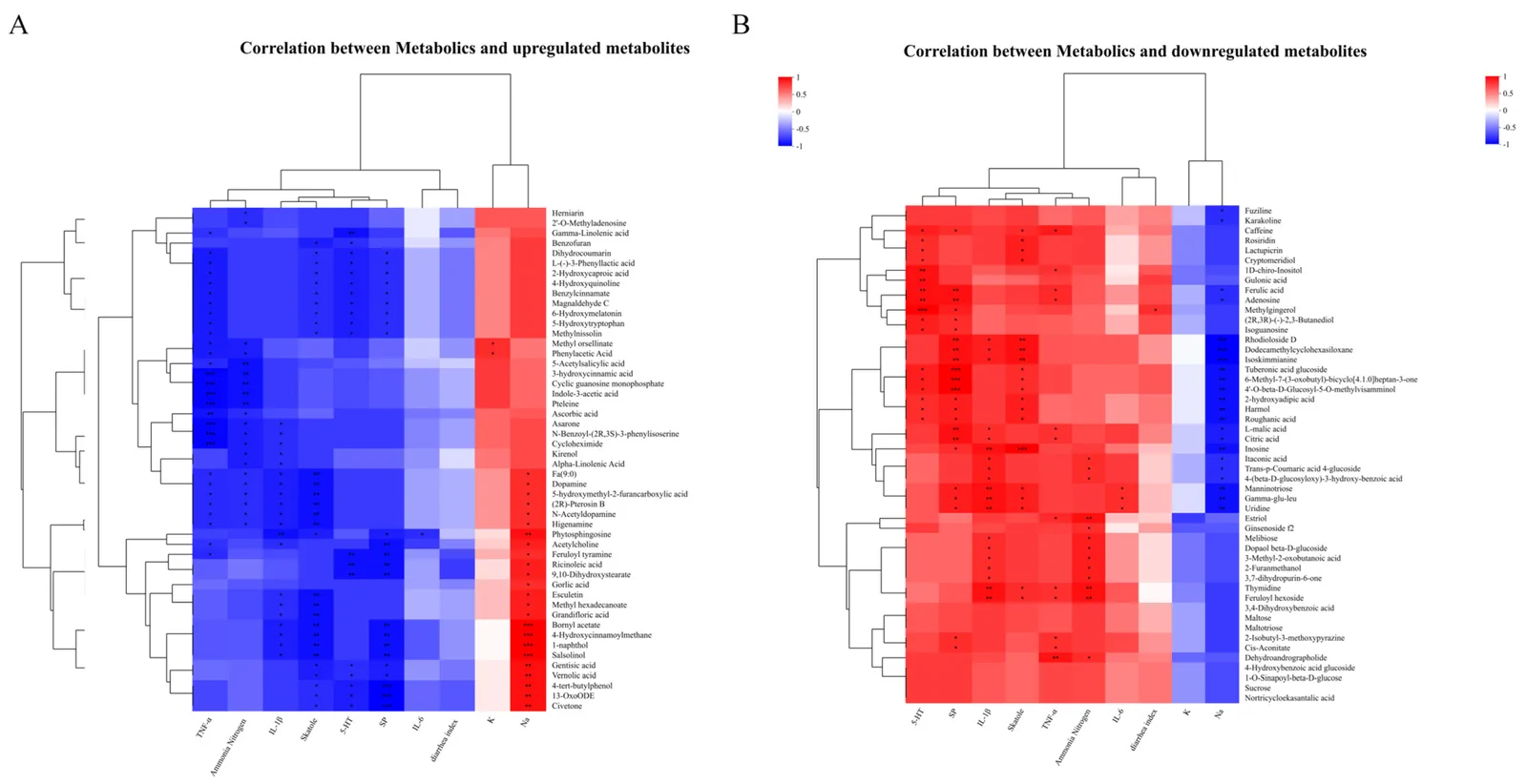
Correlation analysis of differential metabolites with intestinal function and diarrhea-related phenotypic indicators. (**A**) Up-regulated metabolites. (**B**) Down-regulated metabolites.* *p* < 0.05, ** *p* < 0.01, *** *p* < 0.001.

Spearman correlation analysis was performed to examine the relationships between differential metabolites before and after fermentation and serum and intestinal physicochemical indices. The results revealed that up-regulated metabolites in fermented *P. oleracea* were generally positively correlated with diarrhea phenotypic indices, inflammatory markers, and neurotransmitter levels, and negatively correlated with electrolyte ion concentrations; conversely, down-regulated metabolites exhibited the opposite correlation patterns. Among the up-regulated metabolites, lipids and lipid-like molecules—including phytosphingosine, gamma-linolenic acid, and 9,10-dihydroxystearate—were significantly negatively correlated with skatole, inflammatory factors (IL-6, IL-1β, TNF-α), and neurotransmitter indices (5-HT, SP) (*p* < 0.05), and significantly positively correlated with Na^+^ levels (*p* < 0.01). Phenylpropanoids such as esculetin, dihydrocoumarin, and feruloyl tyramine were significantly negatively correlated with skatole (*p* < 0.05) and significantly positively correlated with Na^+^ (*p* < 0.01). Organic acids and their derivatives, including L-(-)-3-phenyllactic acid and 5-hydroxymethyl-2-furancarboxylic acid, were markedly negatively correlated with TNF-α as well as neurotransmitter indices (5-HT, SP) (*p* < 0.05, Figure 7A).

For down-regulated metabolites, organic acids and their derivatives such as cis-aconitate, itaconic acid, and tuberonic acid glucoside were significantly positively correlated with skatole, inflammatory indices (IL-1β, TNF-α), and neurotransmitter SP (*p* < 0.05), and significantly negatively correlated with Na^+^ (*p* < 0.01). Alkaloids including caffeine and harmol were significantly positively correlated with skatole, TNF-α, and neurotransmitters (5-HT, SP) (*p* < 0.05), and significantly negatively correlated with Na^+^ levels (*p* < 0.01). Terpenoids such as estriol and dehydroandrographolide exhibited significant positive correlations with ammonia nitrogen and TNF-α (*p* < 0.05, Figure 7B). Correlation analysis thus demonstrated that fermentation-induced up-regulation of lipids, phenylpropanoids, and organic acids was negatively associated with intestinal inflammatory factors and diarrhea-related indices, and positively associated with electrolyte ion levels. In contrast, the down-regulation of organic acids and their derivatives, alkaloids, and terpenoids displayed the opposite correlation pattern. The differential expression of these key metabolites may serve as a crucial metabolic basis for the intervention of fermented *P. oleracea* in alleviating diarrhea and remodeling the intestinal microenvironment.

### 3.8. Transcriptomic Profiling Reveals Fermented P. oleracea Modulates Key Signaling Pathways

**Figure 8:**
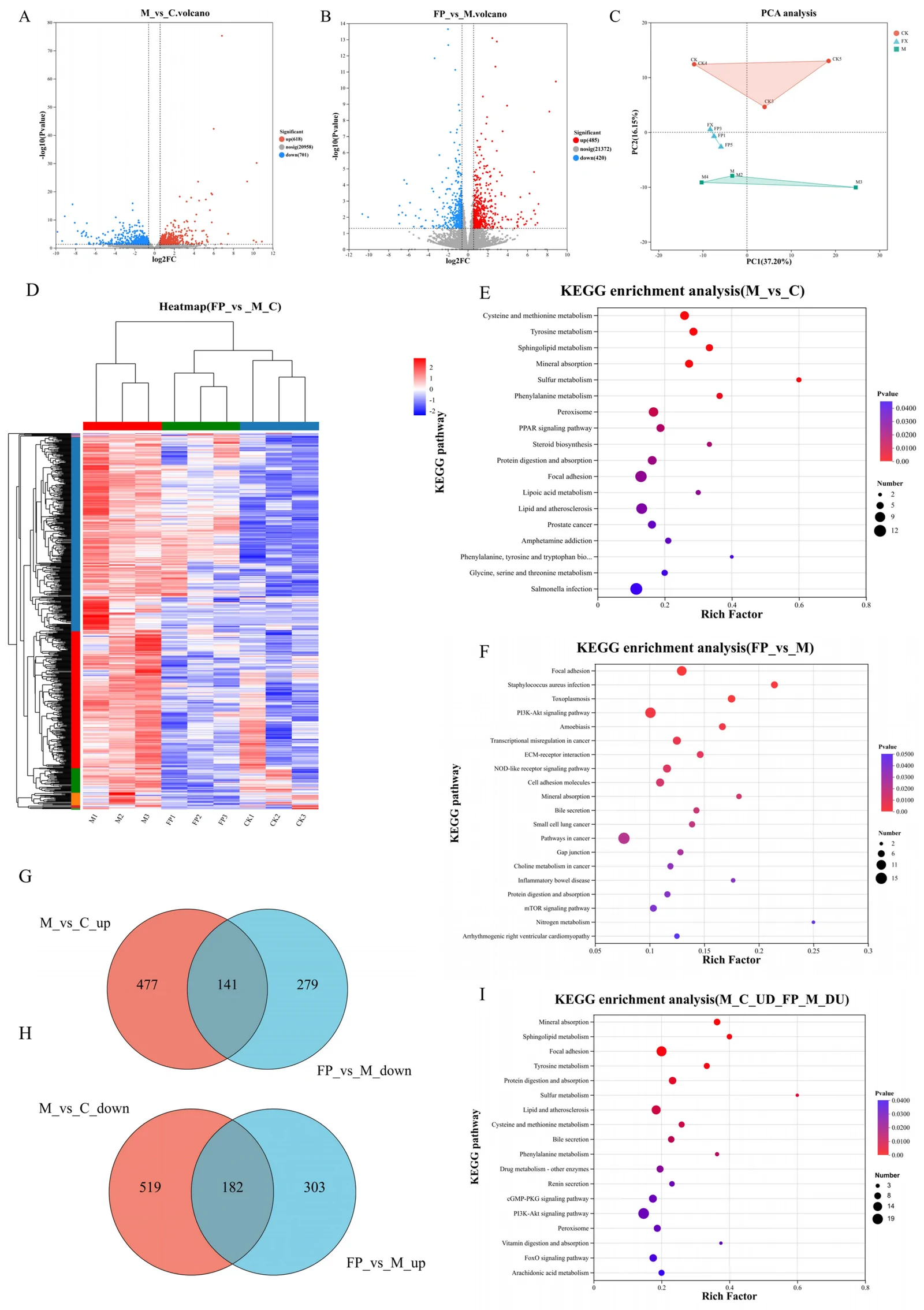
Transcriptomic analysis of ileal tissues reveals key signaling pathways modulated by FP. (**A**,**B**) Volcano plots of differentially expressed genes (DEGs) between control (**C**) and model (M) groups (**A**), and between FP and M groups (**B**). (**C**) Principal component analysis (PCA) plot showing global transcriptomic separation among C, M, and FP groups. (**D**) Heatmap of DEGs across groups; (**E**,**F**) KEGG pathway enrichment analysis of DEGs between C and M groups (**E**) and between FP and M groups (**F**). (**G**,**H**) Venn diagrams showing overlapping DEGs between up- and down-regulated comparisons. (**I**) KEGG enrichment analysis of common DEGs, highlighting pathways related to inflammation, ion homeostasis, and signal transduction.

To elucidate the molecular mechanisms by which FP influences diarrhea in mice, a transcriptomic analysis was conducted. The expression profiles of differentially expressed genes (DEGs) were visualized using volcano plots. Screening criteria included a corrected *p*-value < 0.05 and a |fold change| ≥ 1.5. The results revealed that, in comparison to the control group (C group), the model group (M group) exhibited 701 significantly down-regulated and 618 significantly up-regulated DEGs (Figure 8A). Conversely, the FP group demonstrated 485 up-regulated and 420 down-regulated DEGs relative to the M group (Figure 8B). Principal component analysis (PCA) further distinguished the samples, showing clear separation and clustering between the M group and the FP group, thereby indicating substantial differences between these two groups (Figure 8C). The FP group was distinctly separated from the M group and showed closer clustering to the C group. This observation suggests that FP treatment altered the gene expression profile from resembling that of the M group to more closely aligning with the C group. The hierarchical clustering heatmap of differentially expressed genes (DEGs) demonstrated that the M group possessed a unique gene expression pattern compared to the C group and the FP group, which displayed similar expression profiles (Figure 8D). In the KEGG enrichment analysis, the DEGs between the C group and the M group were significantly enriched across 24 KEGG pathways (*p* < 0.05). These pathways encompassed inflammation-related pathways, such as the Focal adhesion signaling pathway and PPAR signaling pathway; pathways associated with amino acid metabolism and the synthesis of bioactive substances, such as phenylalanine, tyrosine, and tryptophan biosynthesis, and phenylalanine metabolism; as well as pathways related to ion homeostasis, such as mineral absorption (Figure 8E). The DEGs between the M group and the FP group were significantly enriched in 21 KEGG pathways (*p* < 0.05). The identified pathways encompassed those associated with inflammation, including the PI3K-Akt signaling pathway and the focal adhesion signaling pathway, as well as pathways pertinent to ion homeostasis, such as mineral absorption (Figure 8F). Utilizing transcriptomic data and genomic localization annotations, we constructed Venn diagrams to delineate the overlap of differentially expressed genes (DEGs) between the M group and C group (up-regulated), between the FP group and M group (down-regulated), as well as between the M group and C group (down-regulated) and the FP group and M group (up-regulated) (Figure 8G,H). A total of 323 differentially expressed genes (DEGs) were identified as common between the M group and C group, as well as between the FP group and M group. These DEGs were significantly enriched in 21 pathways (*p* < 0.05), including inflammation-related pathways such as Focal adhesion, PI3K-Akt signaling, and FoxO signaling pathways; signal transduction-related pathways such as the cGMP-PKG signaling pathway; and ion homeostasis-related pathways, including mineral absorption and renin secretion (Figure 8I). These findings suggest that FP may mitigate the progression of diarrhea in mice by modulating the focal adhesion, PI3K-Akt signaling, cGMP-PKG signaling, and mineral absorption pathways.

### 3.9. Molecular Validation of Multi-Target Mechanisms

**Figure 9:**
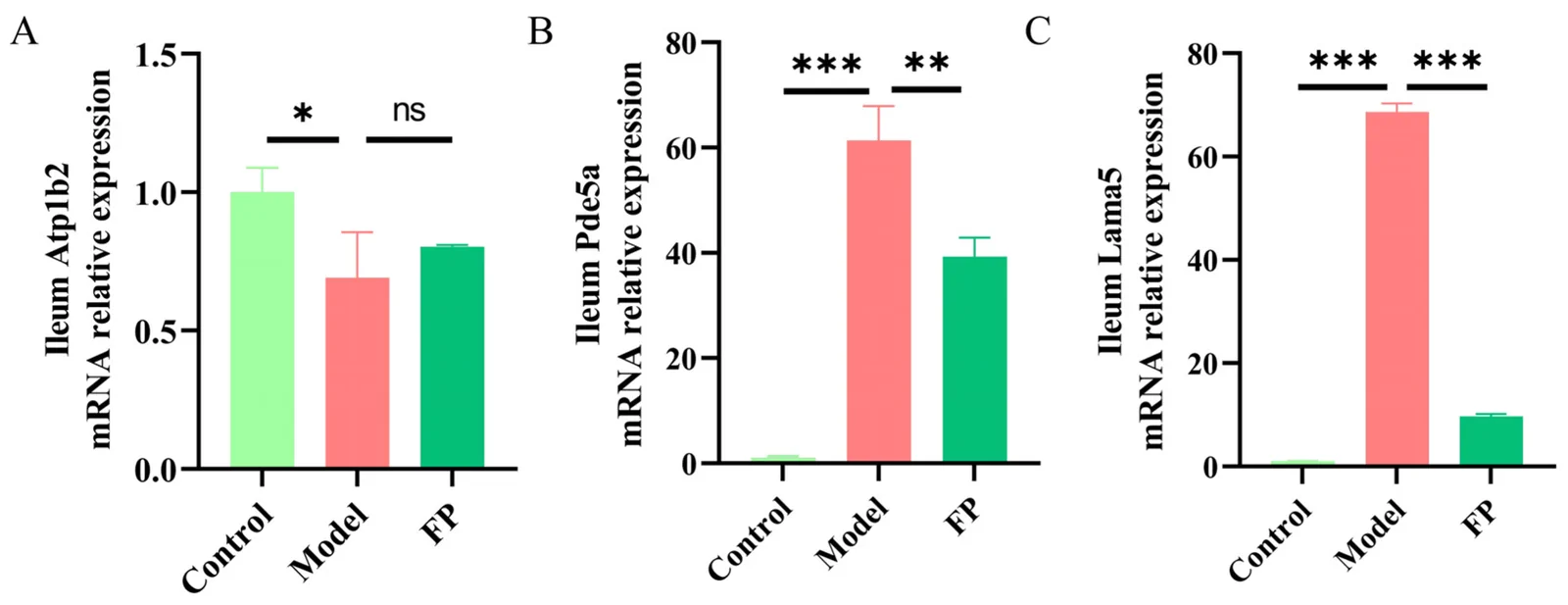
RT-qPCR validation of key differentially expressed genes in the ileum. Relative mRNA expression levels of (**A**) *Lama5*, (**B**) *Pde5a*, and (**C**) *Atp1b2*. Data are expressed as mean ± SD (*n* = 3). * *p* < 0.05, ** *p* < 0.01, *** *p* < 0.001 vs. model group.

Through transcriptomic profiling, candidate genes were identified for validation, and their mRNA expression levels in ileal tissues were quantified using RT-qPCR. The gene *Atp1b2*, which encodes a Na^+^/K^+^-ATPase subunit critical for ion homeostasis, was significantly down-regulated in the senna-induced diarrhea model group, exhibiting a 0.69-fold decrease compared to the control (*p* < 0.05; Figure 9A). Following FP treatment, *Atp1b2* expression increased to 0.80-fold that of the control, although this was not statistically significant compared to the model group. The gene *Pde5a*, associated with neurotransmitter transmission and signal transduction, was significantly up-regulated in the model group, showing a 61.35-fold increase relative to the control (*p* < 0.001, Figure 9B). FP treatment significantly reduced *Pde5a* expression to 39.33-fold of the control (*p* < 0.01), which was notably lower than in the model group. *Lama5*, a gene involved in inflammatory processes, demonstrated a substantial increase in the model group, with a 68.61-fold rise compared to the control (*p* < 0.001, Figure 9C). Fermented *P. oleracea* (FP) treatment significantly reduced its expression to 9.68-fold of the control (*p* < 0.001). RT-qPCR findings validated the transcriptome data, confirming that FP mitigates diarrhea by up-regulating ion balance-related genes (e.g., *Atp1b2*) and down-regulating inflammation and neurotransmitter-related genes (e.g., *Pde5a* and *Lama5*).

## 4. Discussion

*Portulaca oleracea* L. is a globally accepted food–medicine homologous plant with a long history of safe consumption, valued for its heat-clearing, detoxifying, and intestinal health–supporting properties [28]. In a study, a methanolic extract of *P. oleracea* (POE) was prepared, and through the application of UPLC-MS, 85 compounds were identified in the extract, including flavonoids, phenolic acids, alkaloids, organic acids, fatty acids, and terpenoids [29]. Despite its well-documented bioactivity, no comprehensive study has systematically examined the impact of probiotic fermentation on its phytochemical profile—a gap this study aims to address.

In this study, we initially explored the metabolic changes induced by the co-fermentation of *P. oleracea* with *L. plantarum* and *B. subtilis* utilizing an untargeted metabolomics approach, which revealed 691 metabolites in *P. oleracea*. Recent studies indicate that specific microbial strains can modulate bioactive constituents in *P. oleracea*. For instance, fermentation with *L. kutscheri* B7 and *L. plantarum* POM1 increases eugenol content by 400% and enhances linalool levels [13]. Likewise, fermentation with *L. plantarum* JGS49 has been shown to enhance phenylalanine metabolism and promote the accumulation of indole-3-lactic acid, thereby contributing to improved antioxidant activity [30]. In contrast, our findings revealed that co-fermentation with *L. plantarum* and *B. subtilis* significantly altered 220 metabolites, comprising 129 up-regulated and 91 down-regulated. The most pronounced changes were observed in lipid, phenylpropanoid, and polyketide profiles. Specifically, up-regulated metabolites predominantly included lipids (21.60%), followed by phenylpropanoids and polyketides (19.14%). Notably, levels of D-mannose, glucose, and maltose were found to decrease, whereas levels of indole-3-lactic acid, γ-linolenic acid, and phytosphingosine increased, corroborating findings from previous studies [15,30].

In this study, senna leaf, a traditional Chinese medicinal herb, was used to establish a mouse model of acute diarrhea. The principal pathological features observed in this model encompass the disruption of the intestinal mucosal barrier, shortening and atrophy of intestinal villi, disorganization of crypt architecture, and infiltration of inflammatory cells within the intestinal mucosa [31]. Previous research has demonstrated that fermented *P. oleracea* exerts specific anti-inflammatory effects in weaned piglets, significantly reducing serum IL-6 levels on days 14 and 28 post-weaning (*p* < 0.05), while TNF-α, IL-1β, and IL-10 levels remained unchanged [14]. In contrast, this study found that the FP-H treatment significantly decreased serum levels of both IL-6 and TNF-α in mice with acute diarrhea within 4 h, suggesting a potent anti-diarrheal effect.

Senna induces acute diarrhea primarily due to its sennosides, which facilitate the release of bioactive substances, including prostaglandins, histamine, and 5-HT [32]. Additionally, sennosides inhibit the activity of Na^+^/K^+^-ATPase in the intestinal mucosal epithelium, thereby disrupting normal ion and water transport processes within the intestine, ultimately leading to diarrhea [33]. A substantial body of literature suggests that intestinal inflammation stimulates enterochromaffin cells to release excessive 5-HT, subsequently activating sensory neurons to release tachykinins such as substance P [34]. Previous studies have demonstrated that Yupingfeng San alleviates TNBS-induced colitis by reducing the proliferation of enterochromaffin (EC) cells and decreasing 5-HT levels [35]. Furthermore, the aqueous extract of cinnamon has been demonstrated to directly inhibit the expression of colonic 5-HT synthase Tph1 in vivo, thereby modulating 5-HT synthesis and effectively alleviating IBS-D [36]. Furthermore, a previous study using berberine-based nanostructures for IBS-D reported reducing serum 5-HT from 117.56 ng·mL^−1^ to 76.02 ng·mL^−1^ [37]. In this study, FP intervention significantly reduced serum 5-HT levels in model mice from 183.82 ng·mL^−1^ to 113.76 ng·mL^−1^ within 4 h, an effect markedly superior to that of the UFP group. Given that the current study employed an acute diarrhea model with a shorter intervention duration, these results indicated that probiotic-fermented *P. oleracea* exerts a rapid and remarkable regulatory effect on 5-HT disturbance associated with acute diarrhea.

Dysregulated neurotransmitter release significantly disrupts intestinal water and electrolyte homeostasis via the enteric nervous system [38]. 5-HT has the capacity to activate specific 5-HT receptor subtypes on intestinal epithelial cells, leading to the inhibition of sodium ion absorption proteins such as the Na^+^/H^+^ exchanger and the enhancement of chloride channel activity via the cystic fibrosis transmembrane conductance regulator. This mechanism promotes chloride ion secretion and consequently increases intestinal fluid secretion [39]. In the present study, our intervention effectively restored serum K^+^ levels from 3.85 mmol·L^−1^ to approximately 4.78 mmol·L^−1^ and Na^+^ levels from 97.83 mmol·L^−1^ to approximately 118.59 mmol·L^−1^ within a 3 h timeframe in a mouse model of acute diarrhea. By contrast, a previous study on guava leaf extract in an IBS-D rat model reported restoring serum K^+^ levels from 2.71 mmol·L^−1^ to 5.60 mmol·L^−1^ and serum Na^+^ levels from 80.65 mmol·L^−1^ to 96.57 mmol·L^−1^ [40]. The key feature of this work is its focus on the acute pathological context, achieving rapid serum potassium normalization within hours, which offers high clinical relevance for acute diarrhea with rapid electrolyte disturbances.

Correlation analysis between metabolites of fermented *P. oleracea* and diarrhea-related physicochemical indicators showed that three significantly up-regulated metabolites after fermentation, namely phytosphingosine, esculetin and feruloyl tyramine, were negatively correlated with 5-HT, SP, TNF-α, IL-1β, IL-6, diarrhea index, ammonia nitrogen and skatole, while being positively correlated with Na^+^. They are the core functional metabolites of fermented *P. oleracea* in alleviating senna-induced acute diarrhea, and they ameliorate diarrheal pathological damage through multi-dimensional synergistic effects. As an important lipid metabolite, phytosphingosine can maintain the integrity of the intestinal epithelial cell membrane, alleviate intestinal barrier damage, inhibit the production and accumulation of intestinal harmful substances such as ammonia nitrogen and skatole, and coordinately regulate the function of Na^+^/K^+^ transport-related proteins to restore serum electrolyte balance [41,42]. Esculetin exerted dual antioxidant and anti-inflammatory effects, inhibiting the overexpression of pro-inflammatory factors and alleviating inflammatory infiltration and histological damage in the intestinal mucosa [43]. As an active phenylpropanoid compound, feruloyl tyramine can reduce the levels of diarrhea-related neurotransmitters, inhibit abnormal intestinal secretion and peristalsis, and promote the repair of intestinal mucosa [44]. Caffeine was significantly down-regulated after fermentation. Its elevated level would promote the elevation of intestinal inflammatory and oxidative stress indicators, with a potential risk of inducing intestinal tissue injury [45]. Fermented *P. oleracea* metabolites synergistically exert the holistic effects of anti-diarrhea, anti-inflammation, intestinal barrier protection, and correction of electrolyte disturbance. Notably, correlation analysis revealed that Dihydrocoumarin and 9,10-Dihydroxystearate were significantly negatively correlated with diarrheal phenotypic indicators, inflammatory indicators and intestinal neurotransmitter indicators (*p* < 0.05), and significantly positively correlated with electrolyte ion levels (*p* < 0.01). At present, few available studies have reported the functions and mechanisms of these two substances in the regulation of diarrhea and the maintenance of intestinal homeostasis.

Transcriptomic analysis provided molecular evidence supporting the aforementioned mechanisms. The Fermented *Portulaca oleracea* L. intervention effectively reversed the dysregulated intestinal gene expression profile induced by the diarrhea model, steering it towards normalization. KEGG enrichment analysis revealed that the differentially expressed genes were significantly enriched in pathways such as focal adhesion, PI3K-Akt signaling, cGMP-PKG signaling, and mineral absorption. Notably, the focal adhesion and PI3K-Akt signaling pathways are of particular interest due to their central roles in regulating intestinal epithelial cell adhesion and migration, which are crucial for maintaining the integrity of the intestinal barrier. Furthermore, these pathways function as pivotal signaling hubs involved in mediating intestinal inflammation and promoting mucosal repair [46]. Damage to the small intestinal mucosa and aberrant basement membrane remodeling lead to compensatory *Lama5* overexpression. This provides a critical signaling framework enabling FP to restore intestinal structure and suppress inflammation [47].

The cGMP-PKG signaling pathway serves as a pivotal regulatory hub for intestinal motility and inflammation. During diarrheal episodes, increased expression of *Pde5a* leads to enhanced degradation of cGMP, which in turn amplifies 5-HT signal transduction and induces excessive contraction of intestinal smooth muscle [48]. A considerable body of research has established that activation of the mineral absorption pathway is crucial for maintaining intestinal electrolyte balance and regulating the ion transport function of intestinal epithelial cells. This pathway is a primary mechanism for alleviating diarrhea [49]. Diarrhea has been shown to down-regulate the expression of the *Atp1b2* gene within this pathway, thereby diminishing the capacity for intestinal electrolyte reabsorption and leading to an imbalance of potassium and sodium ions [50], corroborating previous findings.

Our multi-omics analysis provides a comprehensive view of the metabolic and transcriptional changes associated with FP treatment. Given that the current findings are based on a single experimental model, further validation in infectious or inflammatory settings—such as DSS-induced colitis or enteropathogenic bacterial infection models—would help establish the broader relevance of FP’s therapeutic effects. Future studies could further validate these findings through targeted functional approaches, including candidate metabolite supplementation or depletion in cell or animal models, pharmacological inhibition or genetic perturbation of key pathway nodes, and phosphorylation-level analyses of downstream effectors. These findings provide a foundational framework for future mechanistic investigations into the bioactive metabolites and pathways that mediate the effects of fermented *P. oleracea*, with the understanding that additional layers of regulation—such as host–microbe interactions—warrant further exploration.

## 5. Conclusions

In summary, probiotic fermentation augments the anti-diarrheal efficacy of *P. oleracea* by modulating inflammatory processes, neurotransmitter signaling, and ion transport, as identified through integrated omics correlation analysis. These results provide systematic candidate mechanistic clues for the intestinal protective effects of fermented purslane, offering a solid scientific foundation for its development as a safe, natural functional food or dietary supplement for intestinal health support and diarrhea management. Future research should prioritize the functional validation of the candidate pathways and bioactive metabolites proposed in this study, as well as their therapeutic potential in clinical contexts.

## Figures and Tables

**Table 1:** Gene Names and Primer Sequences.

Primers	NCBI Gene ID	Forward Primer	Reverse Primer
*Atp1b2* [25]	11932	GGCAGGTGGTTGAGGAGTG	GGGGTATGGTCAGAGACGGT
*Lama5* [26]	16776	GCTGGCGGAGATCCCAATC	GTGTGACGTTGACCTCATTGT
*Pde5a* [27]	242202	CGGCCTACCTGGCATTCTG	GCAAGGTCAAGTAACACCTGATT
*GAPDH*	14447	AGGTCGGTGTGAACGGATTTG	TGTAGACCATGTAGTTGAGGTCA

## Data Availability

The original contributions presented in the study are included in the article, further inquiries can be directed to the corresponding author.

## Abbreviations

The following abbreviations are used in this manuscript:

FP	Fermented *Portulaca oleracea* L.
UFP	Unfermented *Portulaca oleracea* L.
IL-1β	Interleukin-1β
IL-6	Interleukin-6
TNF-α	Tumor Necrosis Factor-alpha
5-HT	5-Hydroxytryptamine
SP	Substance P
Na^+^	Sodium ion
K^+^	Potassium ion
Na^+^/K^+^-ATPase	Sodium/Potassium Adenosine Triphosphatase
